# Moving from disclosure to substance in AI transparency

**DOI:** 10.1016/j.patter.2026.101585

**Published:** 2026-06-12

**Authors:** Huixin Zhan, Jason H. Moore

**Affiliations:** 1 Department of Computer Science and Engineering, New Mexico Institute of Mining and Technology, Socorro, NM, USA; 2 Department of Computational Biomedicine, Cedars-Sinai Medical Center, Los Angeles, CA, USA

## Abstract

AI disclosure mandates have rapidly become standard in scientific publishing, a genuine success of scholarly self-governance. Yet across 10,000 bioRxiv preprints, disclosure tracks only modestly with code and data sharing. The next phase of policy should pair “whether” with “how,” alongside independent transparency requirements.

## Main text

### Introduction

AI tools are rapidly entering scientific workflows,^1^^,^^2^ and journals and funders have responded with remarkable speed: in the span of roughly two years, AI-use disclosure has moved from an ad hoc practice to an expected element of scientific publishing.^3^^,^^4^ This represents a genuine success of scholarly self-governance, as the research community identified a new source of methodological opacity and established norms to address it, and authors have substantively complied.

With this foundation in place, the natural next question is how AI disclosure should evolve to maximize its contribution to open, reproducible science. This opinion offers an empirical starting point for that question. Analyzing 10,000 bioRxiv preprints from 2024 to 2025, we document three observations that jointly motivate a more targeted second generation of disclosure policy. First, disclosure and broader transparency practices (code and data sharing) are only weakly coupled. Second, this coupling does not strengthen when disclosure becomes more granular, a pattern one would expect if disclosure primarily tracked open-science commitment. Third, the coupling patterns differ substantially across fields and across the code vs. data dimensions, consistent with task-specific rather than commitment-driven co-occurrence.

Current disclosure practices can, by themselves, communicate certain aspects of AI use, but they cannot reliably indicate the broader transparency commitments of a study. Clarifying both their capabilities and their limits can help publishers and funders design the next layer of policy with realistic expectations about what disclosure mandates achieve in isolation and what they need to be paired with.

### Illustrative evidence

We analyzed 10,000 bioRxiv preprints from 2024 to 2025 using automated extraction of disclosure statements mentioning common AI tools (e.g., ChatGPT, Copilot, Claude) and transparency indicators such as code and data availability.^5^ Our sample spans 16 fields, including neuroscience, bioinformatics, microbiology, and evolutionary biology. We report effect sizes rather than *p* values, since with 10,000 papers, small effects can attain statistical significance without substantive meaning.

### Key findings

AI disclosure is on the rise. From 2024 to 2025, the share of preprints disclosing any AI use increased from 54% to 58%, and the share with clear disclosure (naming specific tools) rose from 22% to 25%. Code and data sharing also improved: valid code links went from 33% to 37%, and valid data links from 42% to 44%. Transparency is trending upward on every dimension we measure, which we read as direct evidence that recent journal and funder policies are achieving their intended first-order effect.

**Figure 1:**
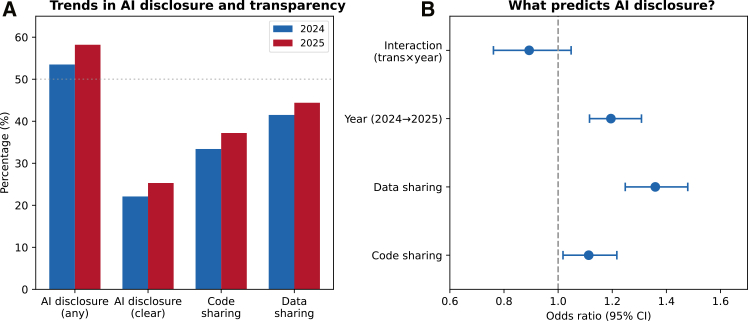
Temporal trends and predictors of AI disclosure (A) Percentages of preprints with AI disclosure (any or clear), code sharing, and data sharing in 2024 vs. 2025. All metrics increased. (B) Forest plot of odds ratios from a multivariable model. Regression estimates illustrate the magnitude of associations rather than implying causal effects. Code sharing and data sharing are associated with higher odds of AI disclosure; the year effect shows diffusion; we find no evidence that the association strengthens over time. In (B), points are odds ratios from multivariable logistic regression, and horizontal error bars indicate 95% confidence intervals; the vertical dashed line marks OR = 1 (no association).

Disclosure and transparency are partially orthogonal. We used logistic regression to estimate the association between transparency practices and AI disclosure. The odds ratio (OR) measures how much more likely disclosure is when a practice is present: OR = 1 means no association; OR = 2 means roughly twice the odds. Papers that share data with a working link have higher odds of disclosing AI use (OR = 1.36, 95% confidence interval [CI] [1.25, 1.48]); papers that share code have modestly higher odds (OR = 1.11, 95% CI [1.02, 1.22]). The 95% CI indicates the range within which the true effect likely lies. These associations are modest because the effect sizes are well below what we would expect if disclosure and transparency were tightly coupled; in that case, OR would typically exceed 2. Here, the associations are real but small, suggesting that disclosure and transparency co-occur only partially. Figure 1 illustrates these patterns.

No evidence indicates temporal strengthening of the association. We evaluated whether the relationship between transparency practices and AI disclosure increased from 2024 to 2025. The estimated interaction term was not distinguishable from zero, and its CI was consistent with no meaningful change over time. We note this as a descriptive observation about the current diffusion trajectory: the coupling between disclosure and sharing is unlikely to strengthen on its own without additional policy design.

Field differences are substantial. Table 1 show that heterogeneity is not random noise but a structured pattern. For data sharing, the strongest associations appear in evolutionary biology (OR = 1.88), cell biology (1.79), developmental biology (1.61), cancer biology (1.58), immunology (1.50), and microbiology (1.49). In contrast, the weakest data-sharing associations are in bioinformatics (1.05), ecology (1.07), molecular biology (1.09), and genomics (1.14). Code-sharing effects are generally smaller and more variable: several fields show near-null or below-1 estimates (e.g., developmental biology 0.96, plant biology 0.89, biophysics 0.72), while only a few fields show strong positive associations (genomics 1.94; immunology 1.89). Taken together, these values support a saturation interpretation in highly codified domains such as bioinformatics: when openness infrastructure is already routine, additional variation in sharing behaviors contributes less to whether AI use is explicitly disclosed.

**Table 1:** Field-specific odds ratios: Association between code/data sharing and AI disclosure

Field	*n*	Code OR	Data OR
Evolutionary biology	634	1.32	1.88
Cell biology	590	1.05	1.79
Developmental biology	328	0.96	1.61
Cancer biology	391	1.14	1.58
Immunology	346	1.89	1.50
Microbiology	703	1.12	1.49
Biophysics	458	0.72	1.48
Plant biology	276	0.89	1.47
Bioengineering	269	1.34	1.22
Neuroscience	2,490	1.19	1.21
Biochemistry	371	1.12	1.15
Genomics	367	1.94	1.14
Molecular biology	354	1.37	1.09
Ecology	422	1.17	1.07
Bioinformatics	938	1.20	1.05

These field differences may reflect broader dynamics documented in the science-of-science literature,^6^ where norms, tools, and transparency practices diffuse unevenly across disciplines. We propose three mechanisms that could explain this heterogeneity. First, baseline transparency saturation: in fields like bioinformatics where code and data sharing are already routine, AI disclosure may be perceived as a procedural requirement—the marginal signal of openness is low. Second, task-specific AI adoption: bioinformatics researchers may use AI primarily for coding, where disclosure norms are less established than for manuscript writing. Third, field-specific openness norms: in evolutionary biology and microbiology, researchers who share data may belong to communities that also embrace disclosure as part of a broader openness ethos. These mechanisms are not mutually exclusive, but they elevate the observation from a descriptive pattern to a theoretically meaningful one. Table 1 provides the full field-level estimates.

### Does more granular disclosure track more strongly with open science?

A natural hypothesis is that researchers most committed to transparency would disclose not merely *that* AI was used, but *how* it was used. Under this hypothesis, the coupling between disclosure and code/data sharing should strengthen as disclosure becomes more substantive. We tested this prediction directly by constructing a four-tier disclosure granularity variable:

Specifically, tier 0 denotes no detectable AI disclosure; tier 1 denotes a general reference (e.g., “a large language model” or “generative AI”) without a named product; tier 2 denotes a named tool (e.g., “ChatGPT” or “Copilot”) without a clear purpose statement in the local text window; and tier 3 denotes a named tool accompanied by a purpose-like phrase in the local window (e.g., “ChatGPT was used to edit the Methods section”).

The tier distribution is itself informative. In the 10,000-paper analytic sample, which corresponds to 97.9% unique URLs, 69.8% (*n* = 6,977) have no detectable AI disclosure, 2.3% (*n* = 232) are tier 1, 25.8% (*n* = 2,575) are tier 2, and only 2.2% (*n* = 216) reach tier 3. Put differently, among papers that disclose AI use at all, roughly 92% stop at naming the tool, and 8% specify its purpose. This pattern, in itself, is a constructive signal to policy designers—current mandates have successfully elicited the minimum required disclosure, and there is substantial headroom for policy to solicit the next layer of detail.

**Figure 2:**
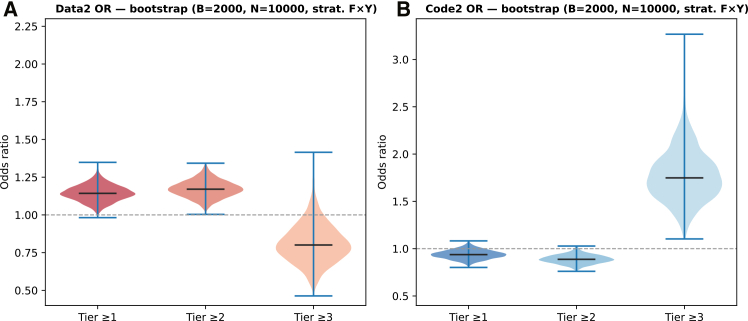
Bootstrap odds ratios for data sharing and code sharing by disclosure tier (A) Data sharing. Bootstrap distributions of odds ratios by disclosure tier. Violin shapes show the distribution of data-sharing odds ratios from B = 2,000 bootstrap resamples (stratified by field × year, *N* = 10,000); the horizontal line in each violin is the median bootstrap OR, vertical whiskers span the minimum and maximum bootstrap values, and the dashed horizontal line at OR = 1 indicates no association. (B) Code sharing. Bootstrap distributions of odds ratios by disclosure tier. Violin shapes show the distribution of code-sharing odds ratios from B = 2,000 bootstrap resamples (stratified by field × year, *N* = 10,000); the horizontal line in each violin is the median bootstrap OR, vertical whiskers span the minimum and maximum bootstrap values, and the dashed horizontal line at OR = 1 indicates no association.

We then estimated the tier-specific association with code and data sharing using logistic regression, and assessed robustness with 2,000 field-by-year stratified bootstrap resamples. The results do not match the pattern predicted by the commitment-driven hypothesis. For data sharing, the OR is 1.14 at tier ≥1, 1.17 at tier ≥2, and 0.80 at tier ≥3; the bootstrap distributions overlap substantially at tiers ≥1 and ≥2 and move *downward*, not upward, at tier ≥3 (Figure 2A). For code sharing, the pattern differs: associations are null or slightly below one at tiers ≥1 and ≥2 (OR 0.94 and 0.89, respectively), then rise sharply to OR ≈ 1.76 at tier ≥3 (Figure 2B), with the bootstrap distribution remaining above one even under resampling uncertainty.

### Building on what works: Four recommendations for the next phase

The findings above should be read in light of what current AI-disclosure policies have already accomplished. In under two years, publishers and funders have established disclosure as a standard element of scientific publishing; most researchers comply, and disclosure rates continue to rise. The policy question is therefore not whether current mandates are working—they are—but how to design their successors. Our data suggest four concrete, empirically grounded directions, each building on rather than replacing existing policy infrastructure.

(1) Move from “whether” to “how.” Our tier distribution shows that current mandates successfully elicit the minimum response (a named tool, 25.8% of papers) but rarely elicit the next layer (purpose statement, 2.2%). Given the strong signal that purpose-specified disclosure carries for certain transparency dimensions (Figure 2B), policy can take a concrete next step by requiring disclosure of four specific elements: tool name and version, stage of use (e.g., writing, programming, data analysis, literature review), scope of use (e.g., whole manuscript, specific sections, specific tasks), and the extent of human review and verification. This recommendation is motivation-neutral: it improves the auditability of AI use regardless of why an author discloses.
(2) Treat disclosure as a parallel requirement, not a proxy. Because the coupling between disclosure and code/data sharing is weak at every tier, policies cannot rely on disclosure mandates to indirectly encourage sharing. Submission checklists should therefore list AI disclosure, code availability, data availability, and method documentation as four independent mandatory items, each with its own reviewer-checkable criterion, rather than bundling them under a single “transparency statement.”
(3) Calibrate to disciplinary baselines. The 15-field heterogeneity in Table 1 shows that a one-size-fits-all policy will not be equally effective everywhere. In fields where code/data sharing is already mature (e.g., bioinformatics), the marginal policy lever is disclosure depth and specificity. In fields where sharing infrastructure is still developing (e.g., cell biology, evolutionary biology), policy should advance disclosure and sharing practices in tandem and provide authors with repository templates and checklists tailored to field conventions.
(4) Evaluate iteratively. The 2024–2025 interaction analysis gives no evidence that disclosure-sharing coupling is strengthening on its own. This is not a verdict on current policy but a design input: if the community’s goal is coupled evolution, policy instruments should be paired with periodic empirical evaluation (every 2–3 years) tracking disclosure rates, disclosure granularity, and coupling strength. If indicators plateau, stronger integrative mechanisms (e.g., making disclosure granularity a reviewer-scored dimension) can be considered.

### Limitations

This study should be interpreted as a conceptual and empirical illustration rather than a definitive causal account. The automated extraction pipeline may under-detect or misclassify disclosure statements, particularly when reporting language is ambiguous or highly field specific. In particular, the tier 3 classifier relies on the co-occurrence of a named tool and a purpose-like phrase within a local window; it may miss cases where purpose is expressed across sentence boundaries or in stylistically unusual ways, and its false-positive rate has not been established against a human-annotated gold standard. In addition, because the analysis is restricted to bioRxiv preprints from 2024 to 2025, the observed patterns may not generalize to peer-reviewed journal corpora, other repositories, or disciplines with distinct publication and transparency norms. Most fundamentally, our observational design cannot distinguish among the several motivations that could generate the disclosure-openness coupling we observe; the policy recommendations above are therefore framed to be valid under any of these interpretations.

## Conclusion

The rapid establishment of AI-disclosure norms across scientific publishing is a genuine achievement of the research community, and our data confirm that these policies are producing their intended first-order effect: disclosure rates are rising. The question we have sought to answer is what the next phase should look like. The presence of AI disclosure, by itself, does not reliably indicate that a paper also shares code or data, and this is true even when disclosure is substantive (names a tool and describes its purpose). This finding has a clear policy implication: if open, reproducible science is the goal, disclosure should be designed as one independent dimension of a multi-dimensional transparency regime, with explicit requirements for how AI was used alongside independent requirements for code, data, and methods. Framed this way, AI disclosure is not a diagnosis of what the community has done wrong—it is the foundation for what the community can build next.

## Acknowledgments

This research is supported by an Institutional Development Award (IDeA) from the National Institute of General Medical Sciences of the National Institutes of Health under grant number P20GM103451. We also acknowledge the bioRxiv team for maintaining open access to preprint metadata and full text. Code for the analysis pipeline is archived at https://doi.org/10.5281/zenodo.19712572.^7^

## Declaration of interests

J.H.M. serves on the advisory board of *Patterns*.

## Declaration of generative AI and AI-assisted technologies in the writing process

The authors used Opus 4.7 to check grammar in the summary and introduction; all content was reviewed and verified by the authors.

## References

[1] Silva T.P., Ocampo T.S.C., Alencar-Palha C., Oliveira-Santos C., Takeshita W.M., Oliveira M.L. (2023). ChatGPT: a tool for scientific writing or a threat to integrity?. Br. J. Radiol..

[2] Ariyaratne S., Iyengar K.P., Botchu R. (2023). Will collaborative publishing with ChatGPT drive academic writing in the future?. Br. J. Surg..

[3] Resnik D.B., Hosseini M. (2025). Disclosing artificial intelligence use in scientific research and publication: When should disclosure be mandatory, optional, or unnecessary?. Account. Res..

[4] Stodden V., McNutt M., Bailey D.H., Deelman E., Gil Y., Hanson B., Heroux M.A., Ioannidis J.P.A., Taufer M. (2016). Enhancing reproducibility for computational methods. Science.

[5] Serghiou S., Contopoulos-Ioannidis D.G., Boyack K.W., Riedel N., Wallach J.D., Ioannidis J.P.A. (2021). Assessment of transparency indicators across the biomedical literature: How open is open?. PLoS Biol..

[6] Cohen-Boulakia S., Belhajjame K., Collin O., Chopard J., Froidevaux C., Gaignard A., Hinsen K., Larmande P., Bras Y.L., Lemoine F. (2017). Scientific workflows for computational reproducibility in the life sciences: Status, challenges and opportunities. Future Gener. Comput. Syst..

[7] Zhan H., Moore J. (2026). From Disclosure to Substance: The Next Step for AI Transparency in Science. Zenodo.

